# Addition of NaCl or Sucrose on the Protein Content, and Functional and Physicochemical Properties of Egg Whites Liquid under Heat Treatment

**DOI:** 10.3390/foods12040881

**Published:** 2023-02-18

**Authors:** Ruihan Yu, Lifeng Wang, Yanqiu Ma, Jingnan Zang, Mingmin Qing, Yujie Chi, Yuan Chi

**Affiliations:** 1 College of Food Science, Northeast Agricultural University, Harbin 150030, China; 2 College of Engineering, Northeast Agricultural University, Harbin 150030, China

**Keywords:** heat treatment, protein content, high-performance liquid chromatography, functional and physicochemical properties

## Abstract

In this study, differences in the protein content and functional and physicochemical properties of four varieties of egg white (EW) were studied by adding 4–10% sucrose or NaCl and then heating them at 70 °C for 3 min. According to a high-performance liquid chromatography (HPLC) analysis, the percentages of ovalbumin, lysozyme and ovotransferrin rose with an increase in the NaCl or sucrose concentration; however, the percentages of ovomucin and ovomucoid decreased. Furthermore, the foaming properties, gel properties, particle size, α-helixes, β-sheets, sulfhydryl groups and disulfide bond content also increased, whereas the content of β-turns and random coils decreased. In addition, the total soluble protein content and functional and physicochemical properties of black bone (BB) chicken and Gu-shi (GS) EWs were higher than those of Hy-Line brown (HY-LINE) and Harbin White (HW) Ews (*p* < 0.05). Subsequently, transmission electron microscopy (TEM) confirmed the changes in the EW protein structure in the four varieties of Ews. As the aggregations increased, the functional and physicochemical properties decreased. The protein content and functional and physicochemical properties of Ews after heating were correlated with the concentration of NaCl and sucrose and the EW varieties.

## 1. Introduction

Egg white protein (EWP) is mainly composed of lysozyme, ovalbumin, ovotransferrin, ovomucin and ovomucoid [1]. With the development of the food industry, the demand for liquid egg whites (Ews) in food production has surged. As a traditional and important food raw material, EW plays an indispensable role in the food industry. Research on the application technology of liquid Ews could improve the processing and production efficiency of the food industry and bring great economic benefits to enterprises [2].

EWP is labile to heat, which can change the function and physicochemical properties of EW after heat treatment [3]. Therefore, pasteurization can be used for EW in industrial production; furthermore, the temperature and heating time of pasteurization are different for different countries, different egg varieties and different feeding modes. In most countries, the heating temperature of liquid EW is in the range 55–58 °C, and the heating time is from 10 s to a few minutes [4]. Pasteurization can retain the nutrition and flavor of EW products well; however, it reduces the quality guarantee period. Higher pasteurization temperatures and heating times could increase the quality guarantee period; however, high temperatures and long heating times could lead to denaturation and aggregation of EWP.

Adding sucrose or NaCl can provide more flavor and heat resistance to EWP in food processing. The dosage of ingredients used in the food industry is generally more than 3%, and the common food ingredients of EW in industrial production are sucrose and NaCl [5,6]. EW with added sucrose and NaCl can have a long quality guarantee period after heat treatment, and the functional and physicochemical properties are less affected [7].

In this study, we took liquid EW and added different concentrations of NaCl and sucrose to examine their effects on the functional properties of different varieties of pasteurized (70 °C, 3 min) Ews, in order to improve the heat resistance of liquid egg white while maintaining its original functional characteristics.

Previous studies have shown that different protein contents of EWP could lead to samples having different functional and physicochemical properties. Moreover, different proteins in EW have different levels of sensitivity to heat treatment, which can increase the quality guarantee period and retain the nutrition of EW [8]. Heat treatment has many advantages, such as its safety, capacity for large batches, simple operation, short reaction time and reduced nutrient loss [5]; therefore, it is the preferred method for processing raw food materials [9].

In this research, we studied the heat treatment of varieties of Ews with 0%, 4%, 6%, 8% and 10% concentrations of NaCl or sucrose. The four varieties of eggs were from the brown Hy-Line variety (HY-LINE) from the United States; the Harbin White (HW) chicken, which is a cross-breed of the European White Leghorn chicken and a native Chinese chicken; and the Gu-shi (GS) chicken and the Black Bone (BB) chicken, which are from China [10]. The five main proteins in EW, supplemented or not with different concentrations of NaCl or sucrose, were analyzed in four varieties of egg whites by high-performance liquid chromatography. The changes in functional, physical and chemical characteristics in different varieties of EW samples were examined. These results would be helpful in selecting EW varieties with high heat resistance and stable functional characteristics, as well as providing theoretical guidance for actual food production and establishing a foundation for further systematic research in the future.

## 2. Materials and Methods

### 2.1. Samples and Reagents

Brown Hy-Line (HY-LINE) eggs, Harbin White (HW) eggs, Gu-shi (GS) eggs and Black Bone (BB) eggs were purchased from a local supermarket. Samples were heated at 70 °C for 3 min, and 4%, 6%, 8% and 10% sucrose or NaCl was added to these samples. We then filtered the impurities in the samples after each round of heating. The chemicals used for HPLC were of HPLC grade; the other chemicals were of analytical grade. Three replicate samples were measured for each treatment.

### 2.2. Total Soluble Protein Content

The different contents of egg white protein (EWP) in the four varieties were studied using ultraviolet spectrophotometry, and the EW samples were measured at 595 nm. For this, 0.5 mL of each EW sample was dissolved in 9.5 mL of a 0.15 mol/L NaCl solution, and then the samples were placed in a centrifuge for centrifugation for 3 min at 10,000× *g* and 20 °C. A 200 mL aliquot of the supernatant was added to 4 mL of Coomassie brilliant blue reagent for 20 min (Coomassie brilliant blue reagent is easy to precipitate and needs to be shaken before use), and the reaction was kept away from the light for 20 min [11].

### 2.3. HPLC Analysis

Next, 0.8 mL of each EW sample was dissolved in sufficient dispersant solution; then the mixture was adjusted to achieve a constant volume of 4 mL and centrifuged at 12,000× *g* for 20 min. Mobile Phase A (Solvent A) contained acetonitrile (90%) and H_2_O (10%); mobile Phase B (Solvent B) contained acetonitrile (10%) and H_2_O (90%). A 50 mL aliquot was loaded onto a Jupiter C4 column (250 mm × 4.6 mm, 300 Å, 5 mm i.d.; Phenomenex, Torrance, CA, USA) maintained at 20 °C, with the detection wavelength at 220 nm [12]. Different proteins were eluted by changing the ratio of Solvent A and Solvent B at a flow rate of 0.8 mL/min. The gradient elution procedure was as follows: for 0–40 min, Solvent A was reduced from 80% to 20%, and Solvent B was increased from 20% to 80%. The running time (including the column equilibrium time) of a single sample was 60 min [13].

### 2.4. Foam Stability and Foaming Capacity

Foaming capacity and foam stability are important functional properties of proteins used in food production, and are key factors affecting the quality of food. The samples were diluted 10 times with a sodium borate solution, and the volume of liquid EW (V_b_) was measured. The liquid EW was homogenized at 13,000 rpm for 1 min, and the volume of foam (V_c_) and the total volume of liquid and foam (V_a_) were measured. After the samples had been heated for 30 min, the volume of foam (V_d_) was measured [14]. The foaming capacity was calculated as foaming capacity = (V_a_ – V_b_)/V_b_, where V_a_ is the total volume of foam and liquid, and V_b_ is the initial volume of the liquid. The foam stability was calculated as foam stability = V_c_/V_d_, where V_c_ is the volume of bubbles after 30 min and V_d_ is the initial volume of the foam.

### 2.5. Gel Properties

In order to study the gel strength and gel hardness in the four varieties under heat treatment, the EW samples were stirred for 5 min at a rotating speed of 50 r/min, and then a 20 mL protein solution of the liquid EW was taken and put into a 25 mL beaker with a diameter of 32 mm, sealed with clingfilm and heated in a 90 °C water-bath for 30 min. The gel was quickly cooled in running water, and then the samples were rested at 4 °C for 24 h before testing [15]. The samples were measured at 25 °C, and the operating conditions of the texture analyzer with a *p*/0.5 probe were as follows: the speed before the test was 5.0 mm/s, the speed after the test was 2.0 mm/s, the pressing distance was 10 mm and the initiation force was 5 g (Stable Micro Systems, Surrey, UK).

### 2.6. Sulfhydryl Group and Disulfide Bond Content

Ellman’s reagent (DTNB) was used for studying the content of the total sulfhydryl groups and surface sulfhydryl groups in the EW samples under heat treatment with different NaCl or sucrose concentrations.

Buffer 1 contained 8 mol/L urea, 0.86 mol/L Tris, 0.09 mol/L Gly and 4 mmol/L EDTA. Buffer 2 contained 10 mmol/L β-mercaptoethanol, 8 mol/L urea, 0.86 mol/L Tris, 0.09 mol/L glycine and 4 m mol/L EDTA.

Approximately 0.5 g of each EW sample was solubilized in 50 mL of buffer 1 (pH 8.0) to measure the content of the surface sulfhydryl groups, and buffer 2 (pH 8.0) was used to measure the content of the total sulfhydryl groups. Next, 5 mL of the sample solution plus 0.1 mL of Ellman’s reagent (4 mg/mL in the same buffer) was added. The samples were reacted for 20 min at room temperature, and then were centrifuged at 6000× *g* for 15 min. The content of the total sulfhydryl groups and the surface sulfhydryl groups was calculated as SH (mmol/L) = (73.53 × A_412_ × E)/F, where F is the content of EWP in the different samples (in mg/mL), A_412_ is the absorbance at 412 nm and E is the dilution factor (2.02/2.00). The content of disulfide bonds in the EW samples was calculated as disulfide bond (SS) = (SH_t_ – SH_s_)/2, where SH_t_ is the content of the total sulfhydryl groups and SHs is the content of the surface sulfhydryl groups [16].

### 2.7. Particle Size and Distribution

For this, 1 mL of each EW sample was added to 9 mL of ultrapure water, and then shaken by a tabletop centrifuge at 5000 r/min until fully dissolved. The EW samples were measured using a MICROTRAC S3500 from the United States; the standby time was 15 min, and the test time was 6 min. The test parameters were set as follows: the refractive index and the refractive index of the powder were 1.460 and 1.330, respectively; the particle size detection range was 0.02–2000 μM [17]. The changes in the particle size of the four varieties of Ews in this research with different NaCl or sucrose concentrations were studied by measuring the changes in the average particle size (D4.3).

### 2.8. Secondary Protein Structures

Samples of the four varieties of liquid Ews with 4–10% concentrations of NaCl or sucrose subjected to heat treatment were spread flat on glass slides for Raman spectrum analysis. The excitation wavelength was set to 532 nm, the excitation power was 50 mW and the scanning range was 400–2000 cm^−1^ [18]. Each scan was 60 s, with 10 rounds of integration and three rounds of accumulation. OMNIC software was used for baseline correction and assignment of the spectral peaks, and the spectral peak intensity of phenylalanine (1002 ± 1) cm^−1^ was used as the normalization factor to process the data [19].

### 2.9. Transmission Electron Microscopy

The microscopic morphology of protein aggregates was observed via transmission electron microscopy (TEM). After diluting the different samples 80 times, we dropped the diluted samples onto the carbon film of the copper mesh and adsorbed them for 1 h. The protein samples on the carbon film of the copper mesh were negatively stained with 4% uranyl acetate for 20 min [20,21]. The prepared samples were dried at room temperature and observed via transmission electron microscopy after drying. The operating voltage was 80 kV.

### 2.10. Statistical Analysis

SPSS 26.0 (SPSS Inc., Chicago, IL, USA) was used in this research. All experiments were repeated three times. The data in the table are expressed as the mean ± standard deviation, according to analysis of variance (ANOVA) and Duncan’s multiple-range test (*p <* 0.05). The figures were plotted using OriginPro 2021 (OriginLab Co., Northampton, MA, USA).

## 3. Results

### 3.1. Total Soluble Protein Content of Four Varieties of Ews under Heat Treatment

**Figure 1:**
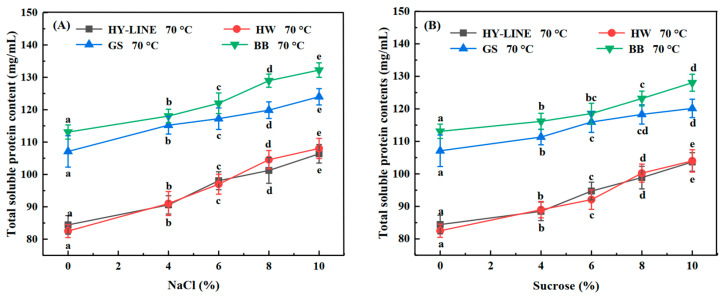
Total soluble protein content of four varieties of EWP subjected to heat treatment. (**A**) Total soluble protein content of four varieties of EWP with different NaCl concentrations after heat treatment. (**B**) Total soluble protein content of four varieties of EWP with different sucrose concentrations after heat treatment. Three replicate samples were measured for each treatment.

The total soluble protein content of the four varieties after heat treatment is shown in Figure 1A,B. The total soluble protein content of EW after heat treatment without NaCl or sucrose was the lowest: HY-LINE, 84.42 mg/mL; HW, 82.54 mg/mL; GS, 107.10 mg/mL; BB, 113.16 mg/mL. After the addition of NaCl or sucrose, the heat resistance of EW increased. The heat resistance and the total soluble protein of the four varieties of Ews with 10% NaCl were the highest. The total soluble protein content of the EW determined the functional and physicochemical properties of the samples [22]. In addition, the total soluble protein content of GS (123.98 mg/mL) and BB (132.25 mg/mL) Ews was significantly higher than that of HY-LINE (106.37 mg/mL) and HW (108.04 mg/mL) Ews (*p* < 0.05), indicating that BB and GS Ews had better foam and gel properties than HY-LINE and HW Ews. EW samples with a high total soluble protein content can form more gel network structures, which can lead to improvements in the gel strength and gel hardness of EW; however, a higher total soluble protein content can promote the foaming capacity and foam stability of liquid EW. In all EW varieties, the different concentrations of NaCl or sucrose in the EW can affect the content of total soluble protein after heat treatment [23]. When the total protein content of the EW increased, the thermostability and functional and physicochemical properties of the EW increased.

### 3.2. The Contents of Different Proteins in Four Varieties of EWs under Heat Treatment

**Figure 2:**
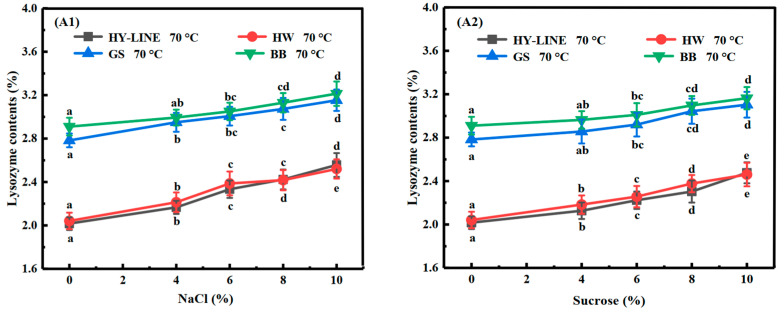

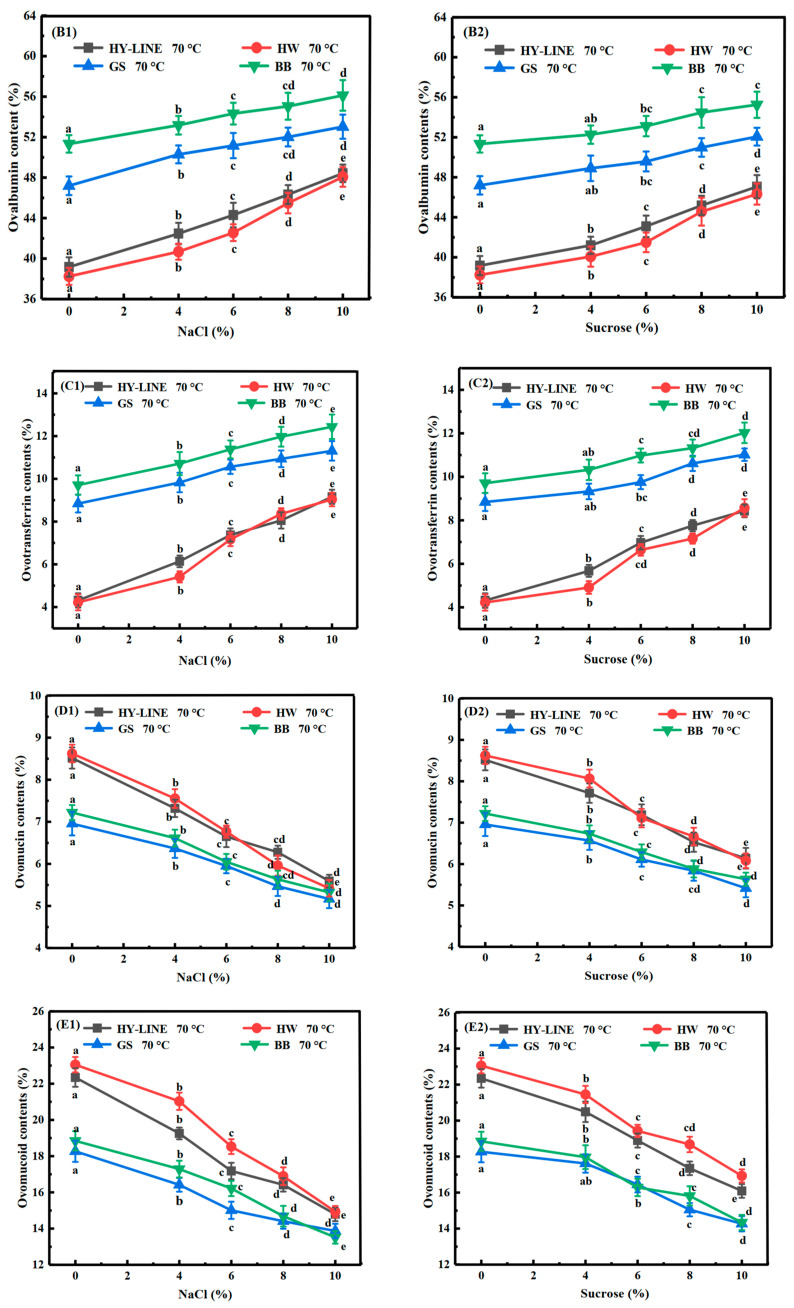
Contents of different proteins in four varieties of EWs subjected to heat treatment. (**A1**) Lysozyme; (**B1**) ovalbumin; (**C1**) ovotransferrin; (**D1**) ovomucin; (**E1**) ovomucoid in four varieties of EW with different NaCl concentrations after heat treatment. (**A2**) Lysozyme; (**B2**) ovalbumin; (**C2**) ovotransferrin; (**D2**) ovomucin; (**E2**) ovomucoid in four varieties of EWs with different sucrose concentrations after heat treatment. Three replicate samples were measured for each treatment.

According to Figure 2(A1,A2), the lysozyme content of EWs without NaCl or sucrose was as follows: HY-LINE, 2.02%; HW, 2.04%; GS, 2.78%; BB, 2.91%. With an increase in the NaCl or sucrose concentration in the EW, the lysozyme content increased. When the NaCl content was 10%, the lysozyme content was the highest: HY-LINE, 2.54%; HW, 2.51%; GS, 3.15%; BB, 3.21%. Lysozyme has antibacterial properties and has an inhibitory effect on Gram-positive bacteria, aerobic spore-forming bacteria, bacillus subtilis and lichen-type bacillus; however, it has no disadvantageous effect on human cells because human cells do not have cell walls [24,25]. Therefore, EW varieties with a high lysozyme content can have better antibacterial properties and a longer quality guarantee period. The results show that the lysozyme content of BB and GS EWs was higher than that of HY-LINE and HW EWs at different concentrations of NaCl or sucrose after heating at 70 °C for 3 min (*p* < 0.05). These results show that the quality guarantee period of BB and GS EWs after heat treatment might be longer than that of HY-LINE and HW EWs.

**Figure 3:**
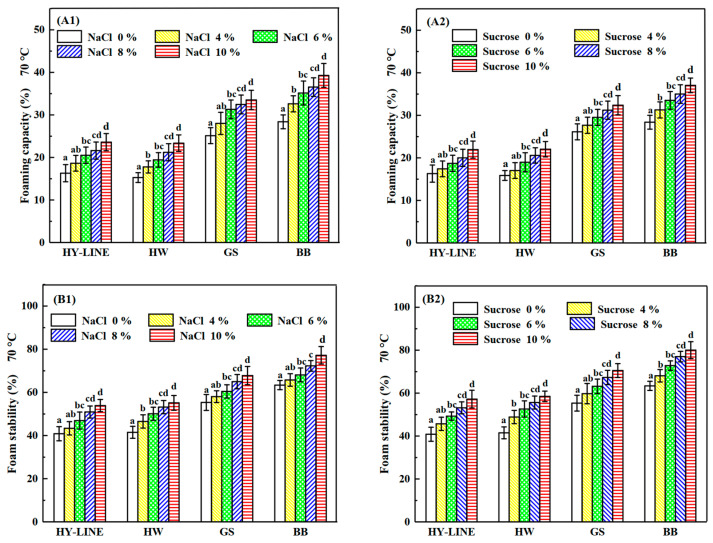
Foaming capacity and foam stability of four varieties of EWP after heat treatment. (**A1**) Foaming capacity of four varieties with different NaCl concentrations after heat treatment. (**A2**) Foaming capacity of four varieties with different sucrose concentrations after heat treatment. (**B1**) Foam stability of four varieties with different NaCl concentrations after heat treatment. (**B2**) Foam stability of four varieties with different sucrose concentrations after heat treatment. Three replicate samples were measured for each treatment.

**Figure 4:**
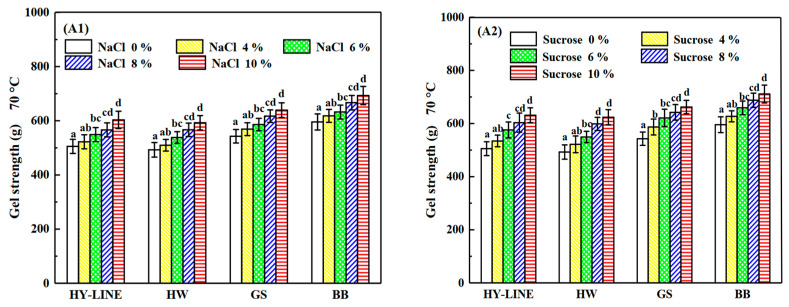

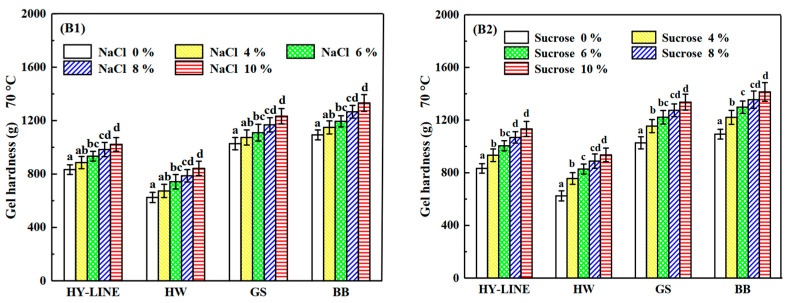
Gel strength and gel hardness of four varieties of EWP after heat treatment. (**A1**) Gel strength of four varieties with different NaCl concentrations after heat treatment. (**A2**) Gel strength of four varieties with different sucrose concentrations after heat treatment. (**B1**) Gel hardness of four varieties with different NaCl concentrations after heat treatment. (**B2**) Gel hardness of four varieties with different sucrose concentrations after heat treatment. Three replicate samples were measured for each treatment.

Ovalbumin determines the foam and gel properties of EW samples [26]. Ovalbumin is widely used in the food industry as a food ingredient because of its favorable functional properties [13]. As shown in Figure 2(B1,B2), the ovalbumin content after heat treatment without NaCl or sucrose was the lowest: HY-LINE, 39.17%; HW, 38.84%; GS, 47.19%; BB, 51.33%. After NaCl or sucrose was added to the EW, with the increase in the concentration of EW, the ovalbumin content increased. The ovalbumin content was the highest for 10% NaCl: HY-LINE, 48.42%; HW, 48.07%; GS, 53.03%; BB, 56.12%. These results indicate that the ovalbumin content might be sensitive to the temperature and the NaCl or sucrose concentrations. The content of ovalbumin in EWs without heat treatment was as follows: HY-LINE, 53.98%; HW, 53.57%; GS, 56.73%; BB, 59.72%. The content of precipitation was as follows: HY-LINE, 14.81%; HW, 14.73%; GS, 9.54%; BB, 8.39%. According to Figure 3 and Figure 4, the foaming capacity, foam stability, gel strength and gel hardness increased when the ovalbumin content increased. Therefore, the ovalbumin content might determine the functional properties of EWs. The ovalbumin content of BB and GS EWs was higher than that of HY-LINE and HW EWs (*p* < 0.05). Therefore, the high ovalbumin content suggests that GS and BB EWs might have better foam and gel properties. The results show that BB and GS EWs were less affected by the heat treatment, and might be more suitable as raw food materials.

The ovotransferrin content of different samples is shown in Figure 2(C1,C2). Additionally, the ovotransferrin content after heat treatment without NaCl or sucrose was the lowest: HY-LINE, 4.31%; HW, 4.22%; GS, 8.84%; BB, 9.71%. After NaCl had been added to the EW, making the concentration reach 10%, the ovotransferrin content was the highest: HY-LINE, 9.16%; HW, 9.03%; GS, 11.35%; BB, 12.43%. Ovotransferrin can interact with iron ions and is susceptible to thermal denaturation; thus, it could be used as an iron supplement [27]. The content of ovotransferrin in EWs without heat treatment was as follows: HY-LINE, 13.24%; HW, 13.11%; GS, 15.02%; BB, 16.18%. The content of precipitation was as follows: HY-LINE, 8.93%; HW, 8.89%; GS, 6.18%; and BB, 6.47%. After heat treatment, the ovotransferrin content decreased significantly, but the decrease was less obvious with increasing concentrations of NaCl and sucrose. The ovotransferrin content in BB and GS EWs was higher than that of HY-LINE and HW EWs after heat treatment (*p* < 0.05); therefore, GS and BB EWs might be more nutritious than HY-LINE and HW EWs.

Ovomucin is a protein with strong thermostability and comprises the main allergen in EW [28]. In Figure 2(D1,D2), the ovomucin content of the four varieties of EW increased after the heat treatment. When the concentration of NaCl was 10%, the ovomucin content was as follows: BB, 5.32%; GS, 5.17%; HY-LINE, 5.59%; HW, 5.42%. For the EW samples without NaCl or sucrose, the ovomucin content of BB (7.22%) and GS EWs (7.96%) was lower than that of HY-LINE (8.52%) and HW EWs (8.62%). The addition of NaCl or sucrose improved the heat resistance of EW. When the liquid EW did not contain NaCl or sucrose, the heat resistance of HY-LINE and HW EWs was poor. The heat-sensitive protein denatures at higher temperatures, resulting in an increase in the percentage of heat-resistant protein. With the addition of 10% NaCl or sucrose, the heat-sensitive protein showed less variability at high temperature, and the percentage of heat-resistant protein decreased. The low ovomucin content of BB and GS EWs indicated that more of the thermosensitive proteins in BB and GS EWs remained after the heat treatment, so the change in the percentage of ovomucin after the heat treatment was smaller than that for HY-LINE and HW EWs.

Ovomucoid has strong thermostability and is the other main allergen in EW, and the ovomucoid content affects the thermostability of EW [28]. According to Figure 2(E1,E2), when 10% NaCl or sucrose was added, the ovomucoid content was as follows: HY-LINE, 14.79%; HW, 14.94%; GS, 13.87%; BB, 13.52%. Moreover, for the EW samples without NaCl or sucrose, the ovomucoid content of HY-LINE (22.34%) and HW EWs (23.05%) was higher than that of GS (18.28%) and BB EWs (18.85%). The reason might be that the thermostability of the protein in EW without NaCl or sucrose was destroyed under the heat treatment, and the content decreased. Ovomucoid, as a thermostable protein, was not destroyed by the heat treatment and its percentage increased.

### 3.3. Differences in the Foaming Properties of Four Varieties of EWs under Heat Treatment

The foaming capacity and foam stability of the four varieties of EWs were affected by different NaCl and sucrose concentrations, as shown in Figure 3. After the heat treatment, the foaming capacity and foam stability decreased; however, the foaming capacity and foam stability of BB and GS EWs were significantly higher than those of HY-LINE and HW EWs (*p* < 0.05). For EWs without NaCl or sucrose, the foaming capacity was as follows: HY-LINE, 16.30%; HW, 15.27%; GS, 25.13%; BB, 28.38%. The values of foam stability were as follows: HY-LINE, 40.84%; HW, 41.51%; GS, 55.32%; BB, 63.38%. According to Figure 3, the foaming capacity of EWs with added NaCl was higher than that of EWs with sucrose; however, the foam stability of EWs with sucrose was higher than that of EWs with NaCl. The foaming capacity of EWs containing 10% NaCl was the highest: HY-LINE, 23.59%; HW, 23.36%; GS, 33.52%; BB, 39.29%. The foam stability of EWs containing 10% sucrose was the highest: HY-LINE, 57.22%; HW, 58.49%; GS, 70.51%; BB, 80.03%. When EW is whipped, air enters the liquid to form a foam of EWP and water [29]. Foams of EW are used in foods such as marshmallows and cakes [30]. The foaming properties of EW are affected by many processing factors. With an increase in the ovalbumin content, the foaming capacity and foam stability of EW were enhanced, whereas adding sucrose or NaCl to EW can improve the foam stability and thermostability of EW [31]. In Figure 2(B1,B2), the ovalbumin content of EWs with different concentrations of NaCl or sucrose was different, and the ovalbumin content of BB and GS EWs was higher than that of the other two varieties (*p* < 0.05), supporting the concept that the ovalbumin content is related to the foaming properties. With an increase in the ovalbumin content, the foaming capacity and foam stability increased.

### 3.4. Differences in the Gel Properties of the Four Varieties of EWs under Heat Treatment

The heat-induced gels of different varieties of EWs are shown in Figure 4. The gel strength of HY-LINE, HW, GS and BB EWs was 505.31 g, 492.68 g, 542.94 g and 595.08 g, respectively. Furthermore, the gel hardness of HY-LINE, HW, GS and BB EWs was 832.98 g, 624.31 g, 1027.41 g and 1093.07 g, respectively. Gel strength and gel hardness are affected by temperature and heating time. Adding NaCl or sucrose increased the heat resistance of EW, and the effect of adding sucrose was better than that of adding NaCl because the viscosity of liquid EW was increased by the addition of sucrose. When the concentration of sucrose in the EWs reached 10%, the gel strength was as follows: HY-LINE, 631.21 g; HW, 623.67 g; GS, 662.11 g; BB, 711.15 g. Gel hardness was recorded as HY-LINE, 1132.98 g; HW, 935.64 g; GS, 1336.74 g; BB, 1413.69 g. Moreover, the gel strength and gel hardness of BB and GS EWs were higher than those of the other two varieties (*p* < 0.05). A disulfide bond is a relatively stable covalent bond, which plays a role in stabilizing the spatial structure of peptide chains in protein molecules [32]. When the disulfide bonds increased, the stability of protein molecules against external factors became greater. The disulfide bonds in the EW heat gel formed a spatial network of protein, which can enhance the elasticity and taste of food [33]. The high content of disulfide bonds in EWs can improve the gel strength and gel hardness. The protein is denatured by heating to form a gel. The gel strength and gel hardness are greatly affected by disulfide bonds, hydrophobic interactions and hydrogen bonds [6].

As shown in Table 1 and Table 2, the disulfide bond content of GS and BB EWP was significantly higher than that in HW and HY-LINE EWP (*p* < 0.05), supporting the finding that gel strength and hardness increase along with an increase in the content of disulfide bonds.

### 3.5. Different Contents of Sulfhydryl Groups in Four Varieties of EWs under Heat Treatment

As shown in Table 1 and Table 2, the content of surface sulfhydryl, total sulfhydryl and disulfide bonds in the four varieties of EWs decreased after the heat treatment. When sucrose or NaCl was added to the EWs, the content of the sulfhydryl group was higher than in the samples without sucrose or NaCl; moreover, the content of the sulfhydryl group increased with the concentration of sucrose and NaCl. The content of surface sulfhydryl and disulfide bonds in BB and GS EWs was higher than that in HY-LINE and HW EWs at different sucrose or NaCl concentrations (*p* < 0.05).

Sulfhydryl groups, also known as hydrogen sulfur groups or thiol groups, are composed of a sulfur atom and a hydrogen atom. Furthermore, the disulfide bond and the sulfhydryl group can be mutually converted. The content of disulfide bonds was positively correlated with the gel strength and gel hardness of EWs. The content of sulfhydryl groups in different EW samples corresponded to the concentration of NaCl or sucrose in the different EW varieties [31]. As shown by the results in Figure 3 and Figure 4, the foaming capacity, foam stability, gel strength and gel hardness of samples with high sulfhydryl group content, such as BB and GS EWs, and with 10% NaCl or sucrose in EW samples were higher than HY-LINE and HW. The higher ovalbumin content of the EW samples could lead to a higher content of the surface sulfhydryl groups and total surface sulfhydryl groups, because ovalbumin contains the sulfhydryl groups and disulfide bonds [34]. According to Figure 2(B1,B2), the ovalbumin content of different samples was consistent with the changes in the sulfhydryl group content. With a decrease of the ovalbumin content, the content of surface sulfhydryl, total sulfhydryl and disulfide bonds decreased.

### 3.6. Different Contents of Secondary Structures in Four Varieties of EWs under Heat Treatment

According to Table 3 and Table 4, when the concentration of NaCl or sucrose in the EWs decreased, the content of α-helixes and β-sheets decreased; conversely, β-turns and random coils increased under the heat treatment. The α-helix and β-sheet content of BB and GS EWP was significantly higher than that of HY-LINE and HW EWP (*p* < 0.05). Furthermore, the content of β-turns and random coils in GS and BB EWP was significantly lower than that in HY-LINE and HW EWP (*p* < 0.05). This refers to the case where the main chain of the polypeptide chain rises in a regular spiral around the central axis. Every 3.6 amino acid residues rise in a spiral, with an upward translation of 0.54 nm, and the direction of the spiral is a right-handed spiral [35]. The carbonyl oxygen of each peptide bond forms a hydrogen bond with the fourth N-H. The direction of the hydrogen bond is basically parallel to the long axis of the helix. All peptide bonds in the peptide chain can form hydrogen bonds. In the β-sheet conformation, the peptide bond’s plane is folded into a zigzag shape, and regular hydrogen bonds are formed between N-H and C=O. The main chains of the adjacent peptide chains are involved in the folding, and all peptide bonds are involved in the formation of interchain hydrogen bonds [36]. The α-helix and β-sheet contain hydrogen bonds, which can improve the heat resistance and functional properties of EW. According to Table 2, the content of α-helixes and β-sheets decreased; conversely, β-turns and random coils increased when the concentration of NaCl and sucrose increased in the EW samples. The content of α-helixes and β-sheets in BB and GS EWP was significantly higher than that in HY-LINE and HW EWP (*p* < 0.05). Furthermore, the content of β-turns and random coils in GS and BB EWP was significantly lower than in HY-LINE and HW EWP (*p* < 0.05). According to Figure 4, the gel strength and gel hardness of BB and GS EWs were significantly higher than those of the other varieties (*p* < 0.05). Therefore, the content of α-helixes and β-sheets was correlated with the content of NaCl or sucrose, and also correlated with EW varieties.

### 3.7. Different Particle Sizes of Four Varieties of EWs under Heat Treatment

**Figure 5:**
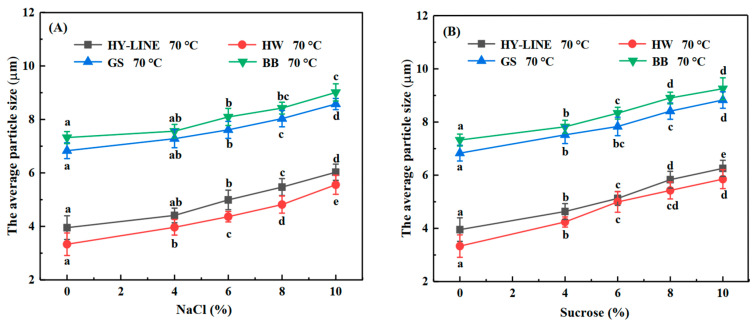
Average particle size of four varieties of EWs after heat treatment. (**A**) Average particle size of four varieties of EWs with different NaCl concentrations after heat treatment. (**B**) Average particle size of four varieties of EWs with different sucrose concentrations after heat treatment. Three replicate samples were measured for each treatment.

The average particle size (D4.3) was sensitive to heat, NaCl and sucrose. The D4.3 of different varieties of EWs is shown in Figure 5. Without NaCl or sucrose, the D4.3 of the EWs is as follows: HY-LINE, 4.05 μm; HW, 3.42 μm; GS, 6.93 μm; BB, 7.42 μm. When the NaCl or sucrose concentration of the EW increased, the D4.3 of the EW increased. When the sucrose concentration of the EW was 10%, the D4.3 was the highest in all four varieties: HY-LINE, 6.26 μm; HW, 5.85 μm; GS, 8.82 μm; BB, 9.25 μm. The size and distribution of particle size are directly related to the food’s industrial processes and product quality [37]. EW samples had aggregations under heat treatment, and the shape of the aggregated protein was inhomogeneous. The error in this analysis can be solved by studying the average particle size. The average particle diameter is the equivalent diameter of the largest particle when the cumulative distribution in the distribution curve of particle size is 50% [38]. When the particle size increases, the gel strength and gel hardness also increase [39]. Furthermore, the differences in the average particle size of different samples support the findings of previous research.

### 3.8. TEM Analysis

**Figure 6:**
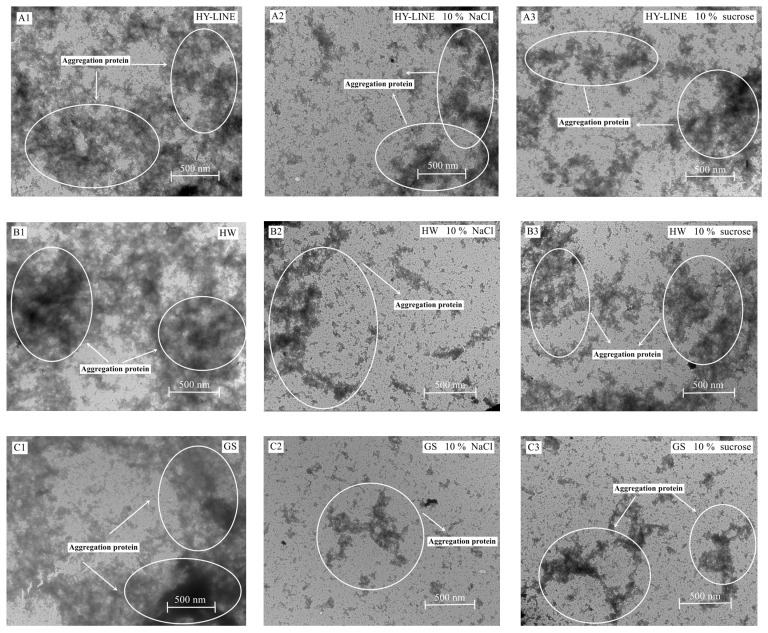

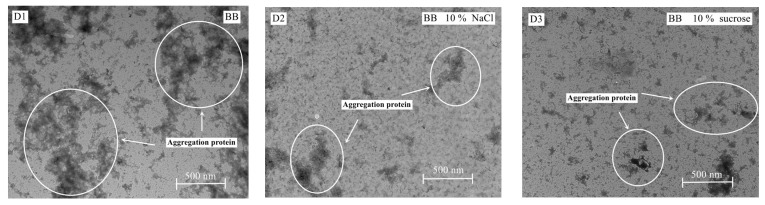
TEM images of four varieties of EWs after heat treatment. (**A1**) HY-LINE EW without NaCl or sucrose after heat treatment. (**A2**) HY-LINE EW with 10% NaCl after heat treatment. (**A3**) HY-LINE EW with 10% sucrose after heat treatment. (**B1**) HW EW without NaCl or sucrose after heat treatment. (**B2**) HW EW with 10% NaCl after heat treatment. (**B3**) HW EW with 10% sucrose after heat treatment. (**C1**) GS EW without NaCl or sucrose after heat treatment. (**C2**) GS EW with 10% NaCl under heat treatment. (**C3**) GS EW with 10% sucrose under heat treatment. (**D1**) BB EW without NaCl or sucrose after heat treatment. (**D2**) BB EW with 10% NaCl after heat treatment. (**D3**) BB EW with 10% sucrose after heat treatment.

As shown in Figure 6, when the area of the aggregation was larger, the heat resistance of the sample was lower [20]. After the addition of NaCl or sucrose, the EWs’ heat resistance increased and the content of thermally polymerized protein decreased; therefore, the level of thermally polymerized protein of BB and GS EWs was significantly lower than that of HY-LINE and HW EWs. TEM is widely used in food science, as the level of thermal polymerization in the EWs after heat treatment can be observed using TEM [21].

The results of TEM suggested that the heat resistance of BB and GS EWs was higher than that of HY-LINE and HW. According to Figure 3 and Figure 4, when the concentration of NaCl or sucrose increased, the foam and gel properties also increased. Moreover, the functional properties of BB and GS EWs were significantly higher than those of HY-LINE and HW EWs (*p* < 0.05). BB and GS EWs had high heat resistance, which suggests that the functional properties of BB and GS EWs were less affected by the heat treatment than the other varieties. Previous results indicate that the state of aggregation is related to the content of α-helixes and β-sheets, and the gel properties. With an increase in the area of the aggregations, the content of α-helixes and β-sheets, and the gel properties, decreased [1,40]. According to Figure 4 and Table 2, the samples with large aggregations had low gel properties and a low content of α-helixes and β-sheets. Small aggregations have high gel properties and a high content of α-helixes and β-sheets. The results of TEM support those of previous studies and suggest that the heat resistance of BB and GS EWs was higher than for HY-LINE and HW EWs.

## 4. Conclusions

The results of this research show that the percentages of lysozyme, ovalbumin and ovotransferrin increased, whereas the percentages of ovomucin and ovomucoid decreased, with an increasing concentration of NaCl or sucrose. The high content of ovomucin and ovomucoid can improve the thermostability of EWs. Furthermore, with an increase in the ovomucin and ovomucoid content in EWs, α-helixes, β-sheets and disulfide bonds increased, which could promote the foaming properties, gel properties and particle size of EW samples. The content of ovomucin, lysozyme, ovalbumin and ovotransferrin in BB and GS EWs was higher than that in HY-LINE and HW EWs. The results show that the functional and physicochemical properties of BB and GS EWs were higher than those of HY-LINE and HW EWs (*p* < 0.05). The protein content of the four varieties was different after the heat treatment, which led to differences in the functional and physicochemical properties. Through TEM observation, for the samples with low thermostability and low functional and physicochemical properties, the aggregation content was high; conversely, the samples with high thermostability and high functional and physicochemical properties had low aggregation content. BB and GS EWs are more suitable for production in the food industry, which provides a theoretical basis for the heat treatment of EWs in the food industry.

## Figures and Tables

**Table 1:** Contents of the surface sulfhydryl groups, total sulfhydryl groups and disulfide bonds of four varieties after addition of NaCl and subejction to heat treatment.

Sulfhydryl Content (mmol/g)	Surface Sulfhydryl	Total Sulfhydryl	Disulfide Bonds
HY-LINE 0% NaCl	1.32 ± 0.08 ^a^	2.32 ± 0.09 ^a^	0.50 ± 0.03 ^a^
HY-LINE 4% NaCl	1.42 ± 0.11 ^b^	2.49 ± 0.11 ^b^	0.54 ± 0.04 ^ab^
HY-LINE 6% NaCl	1.47 ± 0.09 ^b^	2.64 ± 0.14 ^c^	0.59 ± 0.03 ^bc^
HY-LINE 8% NaCl	1.54 ± 0.06 ^c^	2.76 ± 0.13 ^d^	0.61 ± 0.05 ^cd^
HY-LINE 10% NaCl	1.61 ± 0.09 ^d^	2.88 ± 0.11 ^e^	0.64 ± 0.02 ^d^
HW 0% NaCl	1.29 ± 0.09 ^a^	2.27 ± 0.12 ^a^	0.49 ± 0.05 ^a^
HW 4% NaCl	1.41 ± 0.11 ^b^	2.47 ± 0.09 ^b^	0.53 ± 0.03 ^ab^
HW 6% NaCl	1.44 ± 0.10 ^b^	2.59 ± 0.14 ^c^	0.57 ± 0.04 ^bc^
HW 8% NaCl	1.53 ± 0.08 ^c^	2.72 ± 0.10 ^d^	0.60 ± 0.03 ^cd^
HW 10% NaCl	1.60 ± 0.09 ^d^	2.85 ± 0.11 ^e^	0.63 ± 0.05 ^d^
GS 0% NaCl	1.61 ± 0.08 ^a^	2.92 ± 0.09 ^a^	0.66 ± 0.05 ^a^
GS 4% NaCl	1.65 ± 0.06 ^a^	3.04 ± 0.10 ^b^	0.70 ± 0.07 ^ab^
GS 6% NaCl	1.75 ± 0.09 ^b^	3.19 ± 0.07 ^c^	0.73 ± 0.06 ^bc^
GS 8% NaCl	1.80 ± 0.07 ^bc^	3.30 ± 0.12 ^d^	0.75 ± 0.05 ^cd^
GS 10% NaCl	1.84 ± 0.08 ^c^	3.40 ± 0.13 ^e^	0.78 ± 0.06 ^d^
BB 0% NaCl	1.66 ± 0.07 ^a^	3.12 ± 0.10 ^a^	0.73 ± 0.04 ^a^
BB 4% NaCl	1.75 ± 0.06 ^b^	3.29 ± 0.12 ^b^	0.77 ± 0.04 ^ab^
BB 6% NaCl	1.80 ± 0.09 ^b^	3.38 ± 0.11 ^c^	0.79 ± 0.03 ^bc^
BB 8% NaCl	1.93 ± 0.06 ^c^	3.55 ± 0.10 ^d^	0.81 ± 0.05 ^cd^
BB 10% NaCl	1.98 ± 0.08 ^c^	3.66 ± 0.12 ^e^	0.84 ± 0.06 ^d^

Means with different lowercase letters in the same column are significantly different at *p <* 0.05. Data are the means ± standard deviations of three replicates. Three replicate samples were measured for each treatment.

**Table 2:** Contents of the surface sulfhydryl groups, total sulfhydryl groups and disulfide bonds of four varieties after addition of sucrose and subjection to heat treatment.

Sulfhydryl Content (mmol/g)	Surface Sulfhydryl	Total Sulfhydryl	Disulfide Bonds
HY-LINE 0% sucrose	1.32 ± 0.08 ^a^	2.32 ± 0.09 ^a^	0.50 ± 0.03 ^a^
HY-LINE 4% sucrose	1.38 ± 0.05 ^b^	2.41 ± 0.11 ^b^	0.52 ± 0.02 ^ab^
HY-LINE 6% sucrose	1.41 ± 0.07 ^b^	2.53 ± 0.13 ^c^	0.56 ± 0.04 ^bc^
HY-LINE 8% sucrose	1.49 ± 0.11 ^c^	2.67 ± 0.10 ^d^	0.59 ± 0.04 ^cd^
HY-LINE 10% sucrose	1.57 ± 0.12 ^d^	2.80 ± 0.12 ^e^	0.62 ± 0.03 ^d^
HW 0% sucrose	1.29 ± 0.09 ^a^	2.27 ± 0.10 ^a^	0.49 ± 0.05 ^a^
HW 4% sucrose	1.36 ± 0.08 ^b^	2.38 ± 0.13 ^b^	0.51 ± 0.02 ^ab^
HW 6% sucrose	1.42 ± 0.11 ^c^	2.54 ± 0.09 ^c^	0.56 ± 0.06 ^bc^
HW 8% sucrose	1.49 ± 0.10 ^d^	2.67 ± 0.08 ^d^	0.59 ± 0.04 ^cd^
HW 10% sucrose	1.56 ± 0.07 ^e^	2.79 ± 0.11 ^e^	0.62 ± 0.03 ^d^
GS 0% sucrose	1.61 ± 0.08 ^a^	2.92 ± 0.09 ^a^	0.66 ± 0.05 ^a^
GS 4% sucrose	1.63 ± 0.09 ^a^	3.01 ± 0.13 ^b^	0.69 ± 0.06 ^ab^
GS 6% sucrose	1.73 ± 0.06 ^b^	3.17 ± 0.13 ^c^	0.72 ± 0.05 ^bc^
GS 8% sucrose	1.77 ± 0.04 ^c^	3.25 ± 0.11 ^d^	0.74 ± 0.04 ^cd^
GS 10% sucrose	1.82 ± 0.03 ^d^	3.33 ± 0.08 ^e^	0.76 ± 0.07 ^d^
BB 0% sucrose	1.66 ± 0.10 ^a^	3.12 ± 0.10 ^a^	0.73 ± 0.03 ^a^
BB 4% sucrose	1.73 ± 0.12 ^b^	3.25 ± 0.11 ^b^	0.76 ± 0.04 ^ab^
BB 6% sucrose	1.78 ± 0.08 ^c^	3.34 ± 0.13 ^c^	0.78 ± 0.06 ^bc^
BB 8% sucrose	1.88 ± 0.09 ^d^	3.48 ± 0.12 ^d^	0.80 ± 0.03 ^cd^
BB 10% sucrose	1.96 ± 0.07 ^e^	3.62 ± 0.09 ^e^	0.83 ± 0.05 ^d^

Means with different lowercase letters in the same column are significantly different at *p <* 0.05. Data are the means ± standard deviations of three replicates. Three replicate samples were measured for each treatment.

**Table 3:** Content of α-helixes, β-sheets, β-turns and random coils in four varieties after addition of NaCl and subjection to heat treatment.

Secondary Structure Content (%)	α-Helixes	β-Sheets	β-Turns	Random Coils
HY-LINE 0%NaCl	27.96 ± 0.32 ^a^	18.21 ± 0.21 ^a^	32.43 ± 0.43 ^a^	21.40 ± 0.26 ^a^
HY-LINE 4% NaCl	29.43 ± 0.33 ^b^	20.27 ± 0.19 ^b^	30.53 ± 0.36 ^b^	19.77 ± 0.32 ^b^
HY-LINE 6% NaCl	30.60 ± 0.43 ^c^	21.31 ± 0.22 ^c^	29.00 ± 0.41 ^c^	19.09 ± 0.35 ^c^
HY-LINE 8% NaCl	31.94 ± 0.49 ^d^	22.72 ± 0.25 ^d^	26.66 ± 0.26 ^d^	18.68 ± 0.37 ^d^
HY-LINE 10% NaCl	33.08 ± 0.36 ^e^	23.76 ± 0.43 ^e^	25.10 ± 0.58 ^e^	18.06 ± 0.46 ^e^
HW 0%NaCl	27.27 ± 0.31 ^a^	18.02 ± 0.49 ^a^	32.77 ± 0.44 ^a^	21.94 ± 0.32 ^a^
HW 4% NaCl	29.14 ± 0.47 ^b^	20.82 ± 0.42 ^b^	29.66 ± 0.22 ^b^	20.38 ± 0.34 ^b^
HW 6% NaCl	30.31 ± 0.44 ^c^	21.73 ± 0.42 ^c^	28.12 ± 0.33 ^c^	19.84 ± 0.36 ^c^
HW 8% NaCl	31.21 ± 0.39 ^d^	22.64 ± 0.45 ^d^	26.97 ± 0.32 ^d^	19.18 ± 0.25 ^d^
HW 10% NaCl	32.39 ± 0.48 ^e^	23.66 ± 0.52 ^e^	25.22 ± 0.27 ^e^	18.73 ± 0.46 ^e^
GS 0%NaCl	30.95 ± 0.30 ^a^	22.10 ± 0.45 ^a^	27.50 ± 0.23 ^a^	19.45 ± 0.39 ^a^
GS 4% NaCl	32.93 ± 0.46 ^b^	23.38 ± 0.49 ^b^	24.84 ± 0.37 ^b^	18.85 ± 0.20 ^b^
GS 6% NaCl	33.84 ± 0.33 ^c^	24.62 ± 0.33 ^c^	25.61 ± 0.34 ^c^	17.93 ± 0.22 ^c^
GS 8% NaCl	34.18 ± 0.42 ^d^	25.73 ± 0.43 ^d^	22.78 ± 0.29 ^d^	17.31 ± 0.37 ^d^
GS 10% NaCl	35.14 ± 0.37 ^e^	26.27 ± 0.45 ^e^	22.30 ± 0.38 ^e^	16.29 ± 0.46 ^e^
BB 0%NaCl	31.56 ± 0.39 ^a^	23.06 ± 0.45 ^a^	26.56 ± 0.46 ^a^	18.82 ± 0.47 ^a^
BB 4% NaCl	33.53 ± 0.21 ^b^	24.78 ± 0.47 ^b^	24.04 ± 0.43 ^b^	17.65 ± 0.30 ^b^
BB 6% NaCl	34.57 ± 0.37 ^c^	25.72 ± 0.31 ^c^	22.90 ± 0.38 ^c^	16.81 ± 0.33 ^c^
BB 8% NaCl	35.56 ± 0.23 ^d^	26.86 ± 0.41 ^d^	21.37 ± 0.34 ^d^	16.21 ± 0.23 ^d^
BB 10% NaCl	36.25 ± 0.37 ^e^	27.72 ± 0.46 ^e^	20.83 ± 0.24 ^e^	15.20 ± 0.13 ^e^

Means with different lowercase letters in the same column are significantly different at *p <* 0.05. Data are the means ± standard deviations of three replicates. Three replicate samples were measured for each treatment.

**Table 4:** Content of α-helixes, β-sheets, β-turns and random coils in four varieties after addition of sucrose and subjection to heat treatment.

Secondary Structure Content (%)	α-Helixes	β-Sheets	β-Turns	Random Coils
HY-LINE 0% sucrose	27.96 ± 0.32 ^a^	18.21 ± 0.21 ^a^	32.43 ± 0.43 ^a^	21.40 ± 0.26 ^a^
HY-LINE 4% sucrose	29.04 ± 0.37 ^b^	19.82 ± 0.43 ^b^	30.18 ± 0.27 ^b^	20.96 ± 0.37 ^b^
HY-LINE 6% sucrose	29.59 ± 0.36 ^c^	20.96 ± 0.25 ^c^	29.40 ± 0.21 ^c^	20.05 ± 0.41 ^c^
HY-LINE 8% sucrose	31.31 ± 0.43 ^d^	21.27 ± 0.28 ^d^	28.03 ± 0.32 ^d^	19.39 ± 0.33 ^d^
HY-LINE 10% sucrose	32.89 ± 0.33 ^e^	22.03 ± 0.34 ^e^	26.20 ± 0.43 ^e^	18.88 ± 0.39 ^e^
HW 0% sucrose	27.27 ± 0.21 ^a^	18.02 ± 0.49 ^a^	32.77 ± 0.34 ^a^	21.94 ± 0.22 ^a^
HW 4% sucrose	28.84 ± 0.32 ^b^	20.04 ± 0.45 ^b^	29.75 ± 0.32 ^b^	21.37 ± 0.26 ^b^
HW 6% sucrose	29.57 ± 0.35 ^c^	21.18 ± 0.38 ^c^	28.38 ± 0.42 ^c^	20.87 ± 0.27 ^c^
HW 8% sucrose	30.85 ± 0.33 ^d^	22.08 ± 0.29 ^d^	27.34 ± 0.35 ^d^	19.73 ± 0.29 ^d^
HW 10% sucrose	31.92 ± 0.31 ^e^	23.09 ± 0.33 ^e^	25.87 ± 0.29 ^e^	19.12 ± 0.34 ^e^
GS 0% sucrose	30.95 ± 0.20 ^a^	22.10 ± 0.40 ^a^	27.50 ± 0.26 ^a^	19.45 ± 0.29 ^a^
GS 4% sucrose	31.18 ± 0.35 ^b^	23.03 ± 0.33 ^b^	26.76 ± 0.25 ^b^	19.03 ± 0.32 ^a^
GS 6% sucrose	32.07 ± 0.22 ^c^	24.13 ± 0.20 ^c^	25.46 ± 0.33 ^c^	18.34 ± 0.38 ^b^
GS 8% sucrose	33.86 ± 0.38 ^d^	25.22 ± 0.31 ^d^	23.18 ± 0.24 ^d^	17.74 ± 0.29 ^c^
GS 10% sucrose	34.77 ± 0.41 ^e^	25.84 ± 0.29 ^e^	22.73 ± 0.27 ^d^	16.66 ± 0.31 ^d^
BB 0% sucrose	31.56 ± 0.29 ^a^	23.06 ± 0.35 ^a^	26.56 ± 0.26 ^a^	18.82 ± 0.24 ^a^
BB 4% sucrose	32.83 ± 0.37 ^b^	24.19 ± 0.29 ^b^	25.03 ± 0.35 ^b^	17.95 ± 0.27 ^b^
BB 6% sucrose	33.54 ± 0.42 ^c^	25.28 ± 0.33 ^c^	23.61 ± 0.32 ^c^	17.57 ± 0.30 ^c^
BB 8% sucrose	34.79 ± 0.46 ^d^	26.47 ± 0.44 ^d^	21.85 ± 0.29 ^d^	16.89 ± 0.21 ^d^
BB 10% sucrose	35.96 ± 0.32 ^e^	27.05 ± 0.42 ^e^	21.38 ± 0.27 ^e^	15.61 ± 0.17 ^e^

Means with different lowercase letters in the same column are significantly different at *p <* 0.05. Data are the means ± standard deviations of three replicates. Three replicate samples were measured for each treatment.

## Data Availability

Due to the nature of this research, the participants in this study did not agree to their data being shared publicly, so the data in this paper are not available.
